# A systematic review of thromboembolic complications and outcomes in hospitalised COVID-19 patients

**DOI:** 10.1186/s12879-024-09374-1

**Published:** 2024-05-10

**Authors:** Hanies Yuhana Othman, Izzati Abdul Halim Zaki, Mohamad Rodi Isa, Long Chiau Ming, Hanis Hanum Zulkifly

**Affiliations:** 1 Department of Clinical Pharmacy, Fakulti Farmasi, Universiti Teknologi MARA Cawangan Selangor, Kampus Puncak Alam, Bandar Puncak Alam, Selangor Malaysia; 2 Cardiology Therapeutics Research Group, Universiti Teknologi MARA, Puncak Alam, Selangor Malaysia; 3 Faculty of Medicine, Universiti Teknologi MARA Selangor, Sungai Buloh Campus, Sungai Buloh, Selangor Malaysia; 4 School of Medical and Life Sciences, Sunway University, Sunway City, Selangor Malaysia

**Keywords:** Venous thromboembolism, Arterial thromboembolism, Myocardial infarction, Stroke, Deep vein thrombosis, Pulmonary embolism, Mortality

## Abstract

Thromboembolic (TE) complications [myocardial infarction (MI), stroke, deep vein thrombosis (DVT), and pulmonary embolism (PE)] are common causes of mortality in hospitalised COVID-19 patients. Therefore, this review was undertaken to explore the incidence of TE complications and mortality associated with TE complications in hospitalised COVID-19 patients from different studies. A literature search was performed using ScienceDirect and PubMed databases using the MeSH term search strategy of “COVID-19”, “thromboembolic complication”, “venous thromboembolism”, “arterial thromboembolism”, “deep vein thrombosis”, “pulmonary embolism”, “myocardial infarction”, “stroke”, and “mortality”. There were 33 studies included in this review. Studies have revealed that COVID-19 patients tend to develop venous thromboembolism (PE:1.0-40.0% and DVT:0.4-84%) compared to arterial thromboembolism (stroke:0.5-15.2% and MI:0.8-8.7%). Lastly, the all-cause mortality of COVID-19 patients ranged from 4.8 to 63%, whereas the incidence of mortality associated with TE complications was between 5% and 48%. A wide range of incidences of TE complications and mortality associated with TE complications can be seen among hospitalized COVID-19 patients. Therefore, every patient should be assessed for the risk of thromboembolic complications and provided with an appropriate thromboprophylaxis management plan tailored to their individual needs.

## Introduction

By the end of 2019, cases of pneumonia of unknown etiology, believed to have been caused by a new coronavirus named Severe Acute Respiratory Syndrome Coronavirus 2 (SARS-CoV-2) and later known as COVID-19 disease were discovered [1]. The lung epithelium, myocardium, and vascular endothelium are the major sites where the SARS-CoV-2 virus binds to the angiotensin-converting enzyme 2 (ACE2) receptor, which results in lung and cardiovascular complications [2].

Aside from pulmonary complications, cardiovascular complications such as cardiac injury, heart failure, arrhythmia, and atherosclerosis were also reported during the early phases of COVID-19 outbreak [3, 4]. In these cardiovascular complications, endothelial inflammation (endotheliitis) and dysfunction due to the viral infection affected vascular homeostasis and organ perfusion [5]. Endotheliitis is found to be associated with hyperpermeability, vascular endothelial dysfunction, and thrombus formation, eventually resulting in thromboembolic (TE) complications [6].

As more clinical cases emerge, episodes of TE complications, such as arterial thrombosis or venous thrombosis, in hospitalized patients have been widely observed [7]. Deep vein thrombosis (DVT) and pulmonary embolism (PE) characterize venous thromboembolism (VTE) [8], while arterial thromboembolism (ATE) typically manifests as myocardial infarction (MI) or stroke [9].

A cross-sectional study performed in Wuhan, China [10] with a study population of 143 COVID-19 patients reported that approximately half of their hospitalised COVID-19 patients (*n* = 66) developed deep vein thrombosis (DVT). Approximately 35% (*n* = 23/66) of COVID-19-related deaths were observed among those who developed DVT.

A validated mortality prognostic tool identified a few demographic and clinical risk factors, such as age, male sex, hypertension, and obesity, as risk factors for severe disease progression and death in COVID-19 patients [11]. Advanced age is one of the identified mortality risk factors, and it may be due to a high level of reactive oxygen species that could injure vascular endothelial cells and eventually cause TE complications [12].

Furthermore, hospitalized COVID-19 patients with pre-existing comorbidities such as cardiovascular disease, active cancer, diabetes, or a history of TE complications are more likely to be at risk of developing TE complications and mortality [13]. In this review, we aimed to explore the incidence of TE complications in hospitalized COVID-19 patients and the mortality outcomes associated with TE complications from different studies.

## Materials and methods

A literature review was performed using the ScienceDirect and PubMed databases for research articles published. The search strategy was completed using keywords and subject headings related to “COVID-19”, “thromboembolic complications”, “venous thromboembolism”, “arterial thromboembolism”, “deep vein thrombosis”, “pulmonary embolism”, “myocardial infarction”, “stroke”, and “mortality”.

The inclusion criteria of the published articles were based on the study design of observational studies comprising both prospective and retrospective studies that reported on the incidences of TE complications in COVID-19. Meanwhile, the outcome of the studies focused on episodes of venous thromboembolism (deep vein thrombosis and pulmonary embolism) or arterial thromboembolism (myocardial infarction and stroke) and mortality in COVID-19 patients who developed TE complications during hospitalization.

The publication dates included articles published from March 2020 to September 2023. In addition, there was no geographical restriction in the systematic review if the articles were published in English language to ensure the transparency and reliability of all relevant studies. We implemented a SIGN checklist approach to assess the risk of bias in the included study (Table 1).

**Table 1:** Summary risk of bias assessment for each included study based on SIGN checklist: cohort study

Studies		PICO	Section 1: Internal Validity														Section 2: Overall Assessment of the Study			
			S1.1	S1.2	S1.3	S1.4	S1.5	S1.6	S1.7	S1.8	S1.9	S1.10	S1.11	S1.12	S1.13	S1.14	S2.1	S2.2	S2.3	
Klok, Kruip [14]		Yes	Yes	Yes	Yes	Not applicable	Can’t say	Yes	Yes	No	No	Yes	Yes	Can’t say	Yes	Yes	Acceptable	Yes	Yes	
Al-Samkari, Karp Leaf [15]		Yes	Yes	Yes	Yes	Yes	Can’t say	Yes	Yes	Not applicable	No	Yes	Yes	Can’t say	Yes	Yes	Acceptable	Yes	Yes	
Mohamud and Mukhtar [16]		Yes	Yes	Yes	Yes	No	Can’t say	Not applicable	Yes	Not applicable	Can’t say	Yes	Yes	Yes	Yes	Yes	Acceptable	Yes	Yes	
Kaptein, Stals [17]		Yes	Yes	Yes	Yes	No	Can’t say	Not applicable	Yes	Can’t say	Can’t say	Yes	Yes	Can’t say	Yes	Yes	Acceptable	Can’t say	Yes	
Gonzalez-Fajardo, Ansuategui [18]		Yes	Yes	Not applicable	Not applicable	Can’t say	Can’t say	Not applicable	Yes	No	No	Yes	Yes	Yes	Yes	Yes	Acceptable	Yes	Yes	
Jimenez-Guiu, Huici-Sanchez [19]		Yes	Yes	Yes	Yes	Yes	Can’t Say	Yes	Yes	No	No	Yes	Yes	Yes	Yes	Yes	High Quality	Yes	Yes	
García-Ortega, Oscullo [20]		Yes	Yes	Yes	Yes	Not applicable	Can’t Say	Not applicable	Yes	No	No	Yes	Yes	Can’t say	Yes	Yes	Acceptable	Yes	Yes	
Chen, Jiang [21]		Yes	Yes	Yes	Yes	Not applicable	Can’t say	Not applicable	Yes	No	No	Yes	Yes	Yes	Yes	Yes	Acceptable	Yes	Yes	
Martinot, Eyriey [22]		Yes	Yes	Not applicable	Yes	Yes	Can’t say	Yes	Yes	No	Yes	Yes	Yes	Yes	Yes	Yes	Acceptable	Yes	Yes	
Munoz-Rivas, Abad-Motos [23]		Yes	Yes	Not applicable	Yes	Can’t say	Can’t say	Not applicable	Yes	Not applicable	Yes	Yes	Yes	Yes	Yes	Yes	Acceptable	Yes	Yes	
Kajoak, Osman [24]		Yes	Yes	Yes	Yes	No	Can’t say	Not applicable	Yes	Not applicable	No	Yes	Yes	Can’t say	Yes	Yes	Acceptable	Yes	Yes	
Tholin, Fiskvik [25]		Yes	Yes	Yes	Yes	No	Can’t say	Yes	Yes	Not applicable	Can’t say	Yes	Yes	Yes	Yes	Yes	Acceptable	Yes	Yes	
Martínez Chamorro, Revilla Ostolaza [26]		Yes	Yes	Yes	Yes	Can’t Say	Can’t say	Not applicable	Yes	Not applicable	No	Yes	Yes	Can’t say	Yes	Yes	Acceptable	Yes	Yes	
Bozzani, Arici [27]		Yes	Yes	Yes	Not applicable	Not applicable	Can’t say	Not applicable	Yes	Not applicable	Yes	Yes	Yes	Yes	Yes	Yes	Acceptable	Yes	Yes	
Elboushi, Syed [28]		Yes	Yes	Yes	Yes	Can’t Say	Can’t Say	No	Yes	Not applicable	No	Yes	Yes	Yes	Yes	Yes	Acceptable	Yes	Yes	
Rali, O’Corragain [29]		Yes	Yes	Yes	Yes	No	Can’t Say	Not applicable	Yes	Not applicable	No	Yes	Yes	Yes	Yes	Yes	Acceptable	Yes	Yes	
Erben, Franco-Mesa [30]		Yes	Yes	Yes	Yes	No	Can’t Say	Not applicable	Yes	Not applicable	Can’t Say	Yes	Yes	Yes	Yes	Yes	Acceptable	Yes	Yes	
Filippi, Sartori [31]		Yes	Yes	Yes	Yes	Can’t Say	Can’t Say	Not applicable	Yes	Not applicable	Can’t Say	Yes	Yes	Yes	Yes	Yes	Acceptable	Yes	Yes	
Brandao, de Oliveira [32]		Yes	Yes	Yes	Yes	No	Can’t Say	Not applicable	Yes	Not applicable	Can’t Say	Yes	Yes	Yes	Yes	Yes	Acceptable	Yes	Yes	
Haksteen, Hilderink [33]		Yes	Yes	Yes	Yes	Can’t Say	Can’t Say	Not applicable	Yes	Not applicable	Can’t Say	Yes	Yes	Yes	Yes	Yes	Acceptable	Yes	Yes	
Arribalzaga, Martinez-Alfonzo [34]		Yes	Yes	Yes	Yes	Can’t Say	Can’t Say	Not applicable	Yes	Not applicable	Can’t Say	Yes	Yes	Yes	Yes	Yes	Acceptable	Yes	Yes	
Valle, Bonaffini [35]		Yes	Yes	Yes	Yes	No	Can’t Say	Not applicable	Yes	Not applicable	Can’t Say	Yes	Yes	Yes	Yes	Yes	Acceptable	Yes	Yes	
Silva, Jorge [36]		Yes	Yes	Yes	Yes	No	Can’t Say	Not applicable	Yes	Not applicable	Can’t Say	Yes	Yes	Yes	Yes	Yes	Acceptable	Yes	Yes	
Whyte, Kelly [37]		Yes	Yes	Yes	Yes	Can’t Say	Can’t Say	Not applicable	Yes	Not applicable	Can’t Say	Yes	Yes	Yes	Yes	Yes	Acceptable	Yes	Yes	
Vivan, Rigatti [38]		Yes	Yes	Yes	Yes	No	Can’t Say	Not applicable	Yes	Not applicable	No	Yes	Yes	Yes	Yes	Yes	Acceptable	Yes	Yes	
Bruggemann, Spaetgens [39]		Yes	Yes	Yes	Yes	No	Can’t Say	Not applicable	Yes	Yes	Yes	Yes	Yes	Yes	Yes	Yes	Acceptable	Yes	Yes	
Chang, Rockman [40]		Yes	Yes	Yes	Yes	Yes	Can’t Say	Not applicable	Yes	Not applicable	Yes	Yes	Yes	Yes	Yes	Yes	Acceptable	Yes	Yes	
Chaudhary, Padrnos [41]		Yes	Yes	Yes	Yes	Yes	Can’t Say	Not applicable	Yes	No	Yes	Yes	Yes	Yes	Yes	Yes	Acceptable	Yes	Yes	
Fujiwara, Nakajima [42]		Yes	Yes	Not Applicable	Yes	Not Applicable	Can’t Say	Yes	Yes	Not Applicable	Yes	Yes	Yes	Yes	Yes	Yes	Acceptable	Yes	Yes	
Helms, Tacquard [43]		Yes	Yes	Yes	Yes	Yes	Can’t say	Not applicable	Yes	Yes	Yes	Yes	Yes	Yes	Yes	Yes	Acceptable	Yes	Yes	
Lodigiani, Iapichino [44]		Yes	Yes	Not applicable	Yes	Can’t say	Can’t say	Not applicable	Yes	Not applicable	Yes	Yes	Yes	Yes	Yes	Yes	Acceptable	Yes	Yes	
Cueto-Robledo, Navarro-Vergara [45]		Yes	Yes	Not applicable	Yes	Can’t say	Can’t say	Not applicable	Yes	No	Yes	Yes	Yes	Yes	Yes	Yes	Acceptable	Yes	Yes	
Fraissé, Logre [46]		Yes	Yes	Not applicable	Yes	Can’t say	Can’t say	Yes	Yes	Not applicable	Yes	Yes	Yes	Yes	Yes	Yes	Acceptable	Yes	Yes	

Domain:

S1.1: The study addresses an appropriate and clearly focused question

S1.2: The two groups being studied are selected from source populations that are comparable in all respects other than the factor under investigation

S1.3: The study indicates how many of the people asked to take part did so, in each of the groups being studied

S1. 4: The likelihood that some eligible subjects might have the outcome at the time of enrolment is assessed and taken into account in the analysis

S1.5: What percentage of individuals or clusters recruited into each arm of the study dropped out before the study was completed

S1.6: Comparison is made between full participants and those lost to follow up, by exposure status

S1.7: The outcomes are clearly defined

S1.8: The assessment of outcome is made blind to exposure status. If the study is retrospective this may not be applicable

S1.9: Where blinding was not possible, there is some recognition that knowledge of exposure status could have influenced the assessment of outcome

S1.10: The method of assessment of exposure is reliable

S1.11: Evidence from other sources is used to demonstrate that the method of outcome assessment is valid and reliable

S.12: Exposure level or prognostic factor is assessed more than once

S.13: The main potential confounders are identified and taken into account in the design and analysis.

S.14: Have confidence intervals been provided?

S2.1: How well was the study done to minimise the risk of bias or confounding?

S2.2: Considering clinical considerations, your evaluation of the methodology used, and the statistical power of the study, do you think there is clear evidence of an association between exposure and outcome?

S3.3: Are the results of this study directly applicable to the patient group targeted in this guideline?

**Fig. 1:**
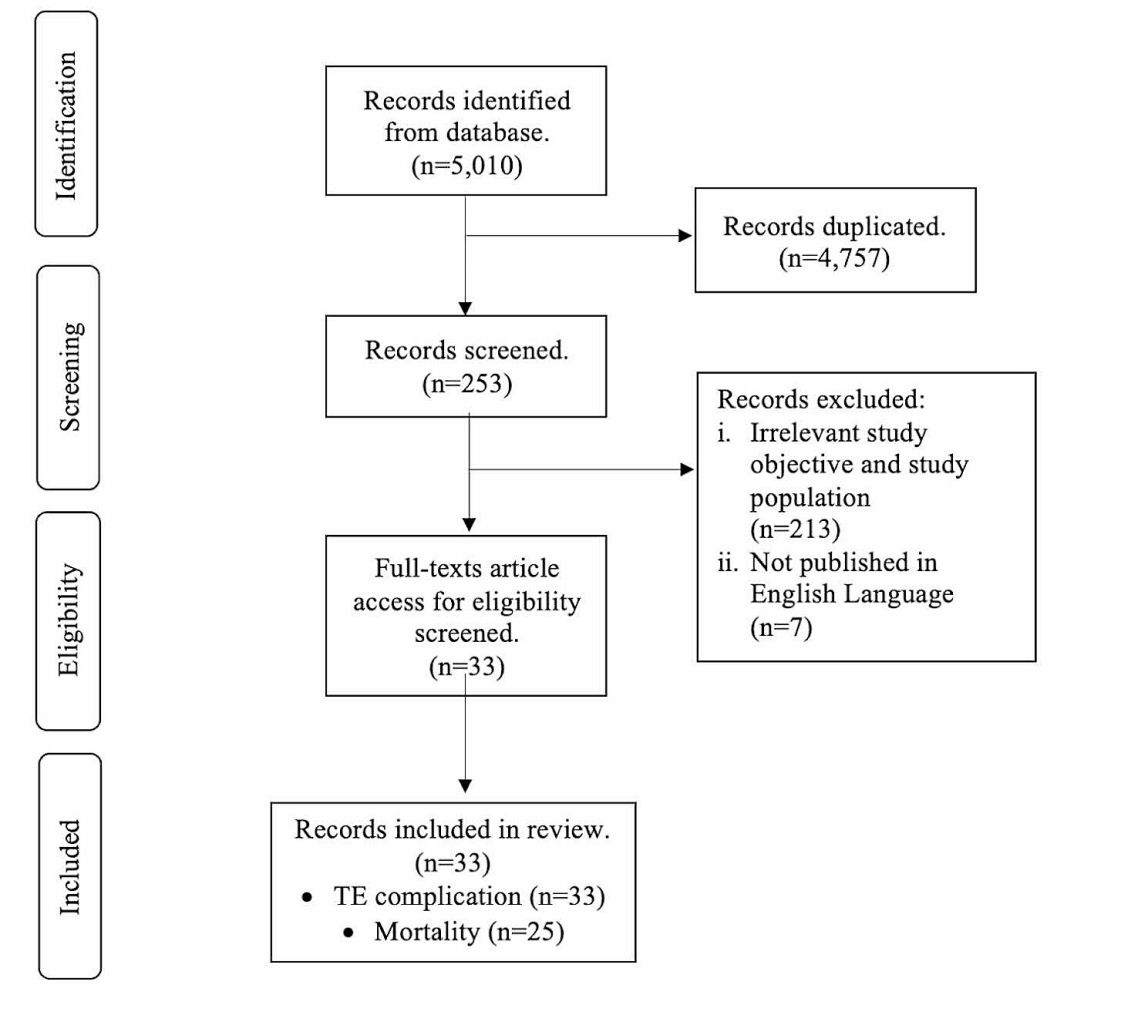
PRISMA flowchart on study selection

The exclusion criteria were all irrelevant study design, topics, and outcomes that were not related to hospitalized COVID-19 patients who developed TE complications. Any duplicate publications, or articles that were not published or accessed in the English language were excluded from the screening and eligibility process (Fig. 1).

Based on the keywords used in the database, we found a total of 5,010 research articles. Finally, after the selection of articles based on the inclusion and exclusion criteria, there were 33 studies included in this review regarding the incidence of TE complications and mortality outcomes among hospitalized COVID-19 patients.

## Results

Most of the included studies have been performed in Europe (*n* = 18) [14, 17–20, 22, 23, 25–27, 33–36, 39, 43, 44, 46], the United States of America (USA) (*n* = 9) [15, 29–32, 38, 40, 41, 45], Asia (*n* = 2) [21, 42], North Africa (*n* = 2) [16, 24] and the United Kingdom [28, 37]. The majority of the studies were conducted retrospectively (*n* = 27) [15–18, 22–42, 44, 46], whereas only six studies were conducted prospectively [14, 19–21, 43, 45]. All study participants were hospitalized with COVID-19 patients, ranging from 23 [21] to 5,966 [34] patients. These studies included patients who were admitted to the intensive care unit (ICU) (*n* = 8) [14, 18, 27, 28, 32, 33], general wards (*n* = 12) [16, 19–21, 29, 30, 35, 36, 38, 40, 41, 45] or a combination of both the ICU and general wards (*n* = 13) [15, 17, 22–26, 31, 34, 37, 39, 42, 44] (Table 2).

**Table 2:** Summary of literature included for epidemiology of TE complications among hospitalised COVID-19 patients

No.	First Author Location	Study Design No. of sample	Study Setting	Venous Thromboembolism				Arterial Thromboembolism	
				PE		DVT		Stroke	MI
1.	Klok, Kruip [14] Netherlands (Europe)	Prospective 184	ICU	13.6% (*n* = 25)		1.6% (*n* = 3)		1.6% (*n* = 3)	**-**
2.	Al-Samkari, Karp Leaf [15] USA	Retrospective 400	ICU and General Ward	2.5% (*n* = 10)		2.3% (*n* = 9)		0.5% (*n* = 2)	2.3% (*n* = 9)
3.	Mohamud and Mukhtar [16] Somalia (North Africa)	Retrospective 46	General ward	13.0% (*n* = 6)		4.3% (*n* = 2)		15.2% (*n* = 7)	8.7% (*n* = 4)
4.	Kaptein, Stals [17] Netherlands (Europe)	Retrospective 947	ICU and General Ward	10.2% (*n* = 97)		0.7% (*n* = 7)		1.3% (*n* = 12)	0.8% (*n* = 8)
5.	Gonzalez-Fajardo, Ansuategui [18] Spain (Europe)	Retrospective 261	ICU	22.2% (*n* = 58)		7.7% (*n* = 20)		5.7% (*n* = 15)	5.0% (*n* = 13)
6.	Jimenez-Guiu, Huici-Sanchez [19] Spain (Europe)	Prospective 57	General ward	**-**		10.5% (*n* = 6)		**-**	**-**
7.	García-Ortega, Oscullo [20] Spain (Europe)	Prospective 73	General ward	35.6% (*n* = 26)		**-**		**-**	**-**
8.	Chen, Jiang [21] China (Asia)	Prospective 23	General ward	**-**		82.6% (*n* = 19)		**-**	**-**
9.	Martinot, Eyriey [22] France (Europe)	Retrospective 600	ICU and General Ward	2.7% (*n* = 16)		2.2% (*n* = 13)		**-**	1.3% (*n* = 8)
10.	Munoz-Rivas, Abad-Motos [23] Spain (Europe)	Retrospective 1127	ICU and General Ward	3.9% (*n* = 44)		0.5% (*n* = 6)		1.2% (*n* = 13)	0.5% (*n* = 6)
11.	Kajoak, Osman [24] Saudi Arabia (North Africa)	Retrospective 445	ICU and General Ward	8.1% (*n* = 36)		**-**		**-**	**-**
12.	Tholin, Fiskvik [25] Norway (Europe)	Retrospective 550	ICU and General Ward	3.6% (*n* = 20)		0.6% (*n* = 3)		0.7% (*n* = 4)	2.0% (*n* = 11)
13.	Martínez Chamorro, Revilla Ostolaza [26] Spain (Europe)	Retrospective 342	ICU and General Ward	26% (*n* = 89)		**-**		**-**	**-**
14.	Bozzani, Arici [27] Italy (Europe)	Retrospective 38	ICU	**-**		84.2% (*n* = 32)		**-**	**-**
15.	Elboushi, Syed [28] UK	Retrospective 198	ICU	14.6% (*n* = 29)		7.6% (*n* = 15)		**-**	**-**
16.	Rali, O’Corragain [29] USA	Retrospective 147	General ward		10.9% (*n* = 16)	9.5% (*n* = 14)		**-**	**-**
17.	Erben, Franco-Mesa [30] USA	Retrospective 915	General ward		9.0% (*n* = 82)			**-**	**-**
18.	Filippi, Sartori [31] USA	Retrospective 267	ICU and General Ward		18.7% (*n* = 50)		**-**	**-**	**-**
19.	Brandao, de Oliveira [32] Brazil (USA)	Retrospective 243	ICU		7.8% (*n* = 19)		3.7% (*n* = 9)	1.2% (*n* = 3)	2.5% (*n* = 6)
20.	Haksteen, Hilderink [33] Netherlands (Europe)	Retrospective 188	ICU		43.1% (*n* = 81)			**-**	**-**
21.	Arribalzaga, Martinez-Alfonzo [34] Spain (Europe)	Retrospective 5966	ICU and General Ward		2.6% (*n* = 176)		0.4% (*n* = 21)	**-**	**-**
22.	Valle, Bonaffini [35] Italy (Europe)	Retrospective 114	General ward		57% (*n* = 65)		**-**	**-**	**-**
23.	Silva, Jorge [36] Portugal (Europe)	Retrospective 300	General ward		15.3% (*n* = 46)		**-**	**-**	**-**
24.	Whyte, Kelly [37] UK	Retrospective 214	ICU and General Ward		37.4% (*n* = 80)		**-**	**-**	**-**
25.	Vivan, Rigatti [38] Brazil (USA)	Retrospective 697	General ward		32.4% (*n* = 226)		**-**	**-**	**-**
26.	Bruggemann, Spaetgens [39] Netherlands (Europe)	Retrospective 60	ICU and General Ward		40.0% (*n* = 24)		**-**	**-**	**-**
27.	Chang, Rockman [40] USA	Retrospective 188	General ward		-		30.9% (*n* = 58)	-	-
28.	Chaudhary, Padrnos [41] USA	Retrospective 102	General ward		1.0% (*n* = 1)		2.9% (*n* = 3)	1.0% (*n* = 1)	2.0% (*n* = 2)
29.	Fujiwara, Nakajima [42] Japan (Asia)	Retrospective 628	ICU and General Ward		1.8% (*n* = 11)		2.1% (*n* = 13)	**-**	**-**
30.	Helms, Tacquard [43] French (Europe)	Prospective 150	ICU		16.7% (*n* = 25)		2.0% (*n* = 3)	0.6% (*n* = 1)	-
31.	Lodigiani, Iapichino [44] Italy (Europe)	Retrospective 388	ICU and General Ward		2.6% (*n* = 10)		1.3% (*n* = 5)	2.3% (*n* = 9)	1.0% (*n* = 4)
32.	Cueto-Robledo, Navarro-Vergara [45] Mexico (North USA)	Prospective 26	General ward		34.6% (*n* = 9)		3.8% (*n* = 1)	3.8% (*n* = 1)	-
33.	Fraissé, Logre [46] French (Europe)	Retrospective 92	ICU		20.1% (*n* = 19)		6.5% (*n* = 6)	2.2% (*n* = 2)	1.1% (*n* = 1)

### Incidence of TE complications in hospitalized COVID-19 patients

Fourteen studies reported both the incidence of VTE (PE and/or DVT) and ATE (stroke and/or MI) in their study population [14–18, 22, 23, 25, 32, 43–47]. Twenty-seven studies [14–18, 20, 22–26, 28, 29, 31, 32, 34–39, 41–46] reported the incidence of PE ranging from 1.0% [41] to 57% [35] with the lowest reported in the USA and the highest in Europe. Patients admitted to the general ward had the highest incidence of PE at 57% [35], followed by those in the ICU and general ward at 40% [39], and those admitted to the ICU alone at 22.2% [18] (Table 2).

Among the 22 studies [14–19, 21–23, 25, 27–29, 32, 34, 40–46] included, DVT was seen among 0.4% (*n* = 21/5966) [34] to 84.2% (*n* = 32/38) [27] of COVID-19 patients seen in European studies. Critically ill patients in the ICU had the highest incidence of 84.2% (*n* = 32/38), followed by 82.6% (*n* = 19/23) in the general ward population [21] and 2.3% (*n* = 9/400) in the combination of the ICU and general ward [15] (Table 1). According to two articles that observed both PE and DVT, the current incidence of VTE was approximately 9.0% (*n* = 82/915) [30] in the COVID-19 population and increased five-fold (*n* = 81/188) [33] in severely ill COVID-19 patients (Table 2).

Twelve studies specifically reported the incidence of both VTE and ATE in their study population [15–18, 23, 25, 32, 41, 43–46]. Although one study [16] showed a higher incidence of ATE [stroke:15.2% and MI:8.7%] than VTE [PE:13.0% and DVT:4.3%], other studies (*n* = 11) showed that VTE was more common than ATE, with the incidence of PE [1.0% [41] to 34.6% [45] and DVT [0.5% [23] to 7.7% [18] compared to stroke [0.5% [15] to 15.2% [16] and MI [0.5% [23] to 8.7% [16].

Fourteen studies reported the incidence of MI and stroke in a hospitalized COVID-19 population [14–18, 22, 23, 25, 32, 41, 43–46]. The incidence of MI ranged from 0.5% (*n* = 6/1127) [23] to 8.7% (*n* = 4/46) [16] with lower rates observed in European studies (0.5-5.0%) [18, 23].

Meanwhile, studies conducted in North Africa, Europe, and the USA revealed that the current incidence of stroke in COVID-19 patients varied between 0.5% (*n* = 2/400) [15] and 15.2% (*n* = 7/46) [16] with Americans having the lowest incidence (0.5–3.8%) [15, 45] (Table 2). Patients admitted to general COVID-19 wards commonly experienced both events, with the North African population having the highest incidences of both stroke and MI (Table 2).

### Outcomes in COVID-19 patients associated with TE complications

In this review, three main outcomes for every hospitalized COVID-19 patient were investigated: discharge, being still hospitalized, and death. To standardize the second outcome in all studies, patients who continued to be in the ICU or general ward, transferred to the general ward from the ICU, or transferred to another hospital were classified as “still hospitalized”.

Eleven articles recorded patients’ discharge status in their studies [14–17, 25, 27, 29, 30, 34, 35, 40]. The number of discharged patients ranged from 12.0% (*n* = 22) [14] to 79.1% (*n* = 22) [34] (Table 2). Patients in the general ward exhibited a higher tendency to discharge (45.7% [16] to 77.2%) [30] compared to those in the ICU [14] to 60.5% [27] (Table 3).

**Table 3:** Outcome for study population

Bil.	Study	No of samples		Study Setting	Discharged	Still Hospitalized	Death
1.	Klok, Kruip [14]	184		ICU	12.0% (*n* = 22)	75.5% (*n* = 139)	12.5% (*n* = 23)
2.	Bozzani, Arici [27]	38		ICU	60.5% (*n* = 23)	15.8% (*n* = 6)	23.7% (*n* = 9)
3.	Elboushi, Syed [28]	198		ICU	-	-	63.1% (*n* = 125)
4.	Brandao, de Oliveira [32]	243		ICU	-	-	31.7% (*n* = 77)
5.	Haksteen, Hilderink [33]	188		ICU	-	-	26.6% (*n* = 50)
6.	Mohamud and Mukhtar [16]	46		General Ward	45.7% (*n* = 21)	39.1% (*n* = 18)	15.2% (*n* = 7)
7.	Valle, Bonaffini [35]	114		General Ward	63.2% (*n* = 72)	21.9% (*n* = 25)	14.9% (*n* = 17)
8.	Silva, Jorge [36]	300		General Ward	-	-	23% (*n* = 69)
9.	Vivan, Rigatti [38]	697		General Ward	-	-	35.6% (*n* = 248)
10.	Chang, Rockman [40]	183		General Ward	43.7% (*n* = 80)	33.3% (*n* = 61)	23.0% (*n* = 42)
11.	Rali, O’Corragain [29]	147		General Ward	49.0% (*n* = 72)	24.5% (*n* = 36)	26.5% (*n* = 39)
12.	Erben, Franco-Mesa [30]	915		General Ward	77.2% (*n* = 707)	13.9% (*n* = 127)	8.9% (*n* = 81)
13.	Bruggemann, Spaetgens [39]	60	ICU and General Ward		-	-	28.3% (*n* = 17)
14.	Filippi, Sartori [31]	267	ICU and General Ward		-	-	17.6% (*n* = 47)
15.	Kaptein, Stals [17]	947	ICU and General Ward		76.2% (*n* = 722)	8.6% (*n* = 81)	15.2% (*n* = 144)
16.	Martinot, Eyriey [22]	600	ICU and General Ward		-	80.8% (*n* = 485)	19.2% (*n* = 115)
17.	Arribalzaga, Martinez-Alfonzo [34]	5966	ICU and General Ward		79.1% (*n* = 4717)	1.1% (*n* = 68)	19.8% (*n* = 1181)
18.	Tholin, Fiskvik [25]	550	ICU and General Ward		40.5% (*n* = 223)	48.4% (*n* = 266)	11.1% (*n* = 61)
19.	Al-Samkari, Karp Leaf [15]	400	ICU and General Ward		55.7% (*n* = 223)	37.0% (*n* = 148)	7.3% (*n* = 29)
20.	Chaudhary, Padrnos [41]	102	ICU and General Ward		-	-	8.8% (*n* = 9)
21.	Fujiwara, Nakajima [42]	628	ICU and General Ward		-	-	4.8% (*n* = 30)

Meanwhile, there were twelve articles [14–17, 22, 25, 27, 29, 30, 34, 35, 40] reported that 1.1% (*n* = 68) [34] to 80.8% (*n* = 485) [22] of their patients remained hospitalized with higher tendencies among those in the ICU compared to patients in the general ward [75.5% [14] vs. 39.1% [16] respectively] (Table 3).

Lastly, 21 studies [14–17, 22, 25, 27–36, 38–42] recorded patients’ death status with incidence ranging from 4.8% (*n* = 30) [42] to 63.1% (*n* = 125) [28]. COVID-19 patients who were critically ill had a higher incidence of mortality [12% (*n* = 23) to 63% (*n* = 125]) than those in the general ward [35.6% (*n* = 248]) (Table 3).

### Incidence of mortality in COVID-19 patients associated with TE complications

Meanwhile, there were 16 studies that reported mortality associated with TE complications [16, 18, 23, 25, 27, 29–31, 33, 35, 36, 38–40, 44, 46]. The incidence of mortality in hospitalised COVID-19 patients due to TE complications ranges from 5.3% [25] to 48.6% [46]. The ICU setting reported the highest incidence, ranging from 23.6% [18] to 48.6% [46]. The general ward has reported a mortality incidence associated with TE complications as high as 42.5% [38] (Table 4).

**Table 4:** Incidence of mortality in COVID-19 patients

Bil.	Study	No of samples	Study Setting	Mortality in hospitalized COVID-19 Patients (Number of mortality cases)	Mortality in hospitalized COVID-19 patients due to TE Complication (Number of mortality from TE / all TE cases)
1.	Klok, Kruip [14]	184	ICU	12.5% (*n* = 23)	-
2.	Gonzalez-Fajardo, Ansuategui [18]	261	ICU	-	23.6% (*n* = 25/106)
3.	Bozzani, Arici [27]	38	ICU	23.7% (*n* = 9)	28.1% (*n* = 9/32)
4.	Elboushi, Syed [28]	198	ICU	63.1% (*n* = 125)	-
5.	Brandao, de Oliveira [32]	243	ICU	31.7% (*n* = 77)	-
6.	Haksteen, Hilderink [33]	188	ICU	26.6% (*n* = 50)	25.9% (*n* = 21/81)
7.	Fraissé, Logre [46]	92	ICU	41.3% (*n* = 38)	48.6% (*n* = 18/37)
8.	Mohamud and Mukhtar [16]	46	General ward	15.2% (*n* = 7)	31.6% (*n* = 6/19)
9.	Rali, O’Corragain [29]	147	General ward	26.5% (*n* = 39)	40.0% (*n* = 12/30)
10.	Erben, Franco-Mesa [30]	915	General ward	8.9% (*n* = 81)	15.9% (*n* = 13/82)
11.	Valle, Bonaffini [35]	114	General ward	14.9% (*n* = 17)	16.9% (*n* = 11/65)
12.	Silva, Jorge [36]	300	General ward	23.0% (*n* = 69)	26.1% (*n* = 12/46)
13.	Vivan, Rigatti [38]	697	General ward	35.6% (*n* = 248)	42.5% (*n* = 96/226)
14.	Chang, Rockman [40]	183	General ward	23.0% (*n* = 42)	19.0% (*n* = 11/58)
15.	Chaudhary, Padrnos [41]	102	General ward	8.8% (*n* = 9)	-
16.	Al-Samkari, Karp Leaf [15]	400	ICU and General Ward	7.3% (*n* = 29)	-
17.	Martinot, Eyriey [22]	600	ICU and General Ward	19.2% (*n* = 115)	-
18.	Munoz-Rivas, Abad-Motos [23]	1127	ICU and General Ward	-	15.9% (*n* = 11/69)
19.	Tholin, Fiskvik [25]	550	ICU and General Ward	11.1% (*n* = 61)	5.3% (*n* = 2/38)
20.	Arribalzaga, Martinez-Alfonzo [34]	5966	ICU and General Ward	19.8% (*n* = 1181)	-
21.	Bruggemann, Spaetgens [39]	60	ICU and General Ward	28.3% (*n* = 17)	37.5% (*n* = 9/24)
22.	Filippi, Sartori [31]	267	ICU and General Ward	17.6% (*n* = 47)	24.0% (*n* = 12/50)
23.	Fujiwara, Nakajima [42]	628	ICU and General Ward	4.8% (*n* = 30)	-
24.	Lodigiani, Iapichino [44]	388	ICU and General Ward	-	25.0% (*n* = 7/28)
25.	Kaptein, Stals [17]	947	ICU and General Ward	15.2% (*n* = 144)	-

## Discussion

In this study, the incidence of TE complications and mortality associated with TE complications in hospitalized COVID-19 patients from European, American, African, and Asian populations were reviewed. The findings showed that hospitalised COVID-19 patients had a high tendency to develop TE complications, which could lead to increased mortality, especially in severely ill patients.

A wide range of TE complications can be seen especially PE (1.0-57%) [35, 41] and DVT (0.4-84.2%) [27, 34] due to large differences in populations across the studies. Although the number of VTE cases reported was relatively comparable with that in other studies, the limited number of patients tended to overestimate the episodes of VTE complications, as the overall cases were summarized in percentage. Hence, studies with small sample sizes tend to report a high incidence of VTE complications [20, 21, 27, 35, 39, 45]. Furthermore, differences in definitions in each study may account for the discrepancy in the incidence of TE complications. For example, a study conducted in the Netherlands [33] reported the incidence of general VTE complications instead of categorizing each VTE event, resulting in an elevated rate of VTE (43.1%, *n* = 81/188).

Moreover, the methods used to diagnose TE complications in each study varied, which could lead to wide variability in the reported incidences. A study [15] found that attending clinicians could not confirm some presumed cases of VTE without clinical evidence consistent with VTE and strong clinical suspicion. This is due to the inability to perform the necessary tests secondary to the diagnostic limitations imposed by the COVID-19 infection.

Aside from that, the wide variation in the incidence of TE complications among hospitalized COVID-19 patients may be due to the absence of a diagnosis for asymptomatic patients, which limits the amount of data collected globally [48]. Moreover, the high number of patients admitted during the COVID-19 pandemic era led to a limited screening for TE complications throughout their hospitalization period. Moreover, two European studies [49, 50] reported that hospital acquired VTE still occurred within 42 days post-discharge and may indicate that some VTE remains undetected, especially in asymptomatic patients.

Similarly, a Dutch study observed no screening for TE complications during admission, unless the patient had a clinical suspicion [17]. As a result, some TE complications remain undiagnosed. These observations were supported by autopsy findings, in which nearly half of the patients (*n* = 11/26) had TE complications, although it was not suspected prior to post-mortem [51]. Therefore, we may underestimate the actual number of TE complications among hospitalized COVID-19 patients.

It was observed that COVID-19 patients in general populations were more likely to develop VTE as compared to ATE complications [14–19, 21–46, 52]. This is explained by the characteristics of the vein, with low pressure owing to the vessel structure and low velocity owing to blood movement against gravity [53]. Most hospitalized COVID-19 patients were either bedridden or isolated in their designated wards. Therefore, restricting their movement and slowing blood flow in veins results in low oxygen tension in the venous wall and a cellular response to initiate inflammation-like TE complications [54, 55].

This review included seven studies, where the patient outcomes varied depending on the study settings: ICU or general ward: ICU or general ward [14, 16, 27, 29, 30, 35, 40]. Patients in the general ward had a higher tendency to be discharged [45.7% [16] to 77.2% [30] than those in the ICU [12.0% [14] to 60.5% [27]. In addition, the incidence of COVID-19 patients who remained hospitalized was also higher among patients in the ICU [15.8% [27] to 75.5% [14] than among those in the general ward [13.9% [30] to 39.1% [16]. This finding is consistent with that of a previous study [9] which showed a higher risk of VTE in the critically ill population due to pre-existing comorbidities and risk factors such as active cancer and a previous history of venous thromboembolism compared to those in the general ward.

ICU patients were more likely to experience all-cause mortality [63.1% [28] vs. 35.6%] [38]. Similarly, ICU patients also had the highest incidence of TE complication-related mortality compared to the other two wards: the general ward and the combination ward [48.6% [46] vs. 42.5% [38] and 37.5% [39]]. The difference in mortality rates in these studies may be related to the patients’ disease prognosis. Critically ill patients are more likely to become hypercoagulable because they can’t move, use mechanical ventilation, or have nutritional deficiencies compared to patients in the medical ward. This exposed them to a higher risk of mortality [56]. Our findings suggest that regardless of the condition of the patients during hospitalization, TE complications in hospitalized COVID-19 patients could lead to poor disease prognosis, thereby increasing patient morbidity and mortality.

By recognizing the incidence of TE complications and mortality in the articles, we gain insight into the burden of TE complications among COVID-19 patients and observe their management across different studies. Most studies [14, 15, 17, 18, 23, 26, 28–39, 41, 42, 44, 46, 57] reported administering thromboprophylaxis to their hospitalized COVID-19 patients. Upon recognition of TE complications, patients received a therapeutic dose of anticoagulant in the absence of prophylactic management [16, 34, 40, 58]. Due to the unknown extent of COVID-19 infection on TE complications at the time, most practitioners had to outweigh the risk and benefit of introducing thromboprophylaxis strategies, either anticoagulants or antiplatelet agents, to hospitalized COVID-19 patients [59] (Table 5).

**Table 5:** Comparison in prophylaxis strategy

No.	First Author Location	Preventive and Manamegent Strategy		No anticoagulant
		Prophylaxis	Therapeutic	
1	Klok, Kruip [10]	All patients received at least standard doses thromboprophylaxis.	-	-
2	Al-Samkari, Karp Leaf [28]	All patients received standard dose thromboprophylaxis.	-	-
3	Mohamud and Mukhtar [39]	-	Only patients with thromboembolic event administered with therapeutic anticoagulant.	Most patients did not receive any prophylaxis.
4	Kaptein, Stals [11]	All patients received standard dose thromboprophylaxis	-	-
5	Gonzalez-Fajardo, Ansuategui [12]	33 patients (31.13%) were treated with thromboprophylaxis. (Low Molecular Weight Heparin)	-	Most patients did not receive any prophylaxis.
6	Munoz-Rivas, Abad-Motos [16]	All patients received standard dose thromboprophylaxis.	Only patients with confirmed TE complications given therapeutic dose.	-
7	Tholin, Fiskvik [17]	Most patients received thromboprophylaxis (61%).	-	-
8	Martínez Chamorro, Revilla Ostolaza [18]	All patients received prophylaxis. (Enoxaparin)	-	-
9	Elboushi, Syed [41]	All patients received prophylaxis. (Low Molecular Weight Heparin)	-	-
10	Rali, O’Corragain [29]	All patients received dose of thromboprophylaxis.	-	-
11	Erben, Franco-Mesa [30]	All patients received standard dose thromboprophylaxis. (Heparin)	-	-
12	Filippi, Sartori [31] USA	Most patients given thromboprophylaxis. (Low Molecular Weight Heparin)	-	-
13	Brandao, de Oliveira [32]	Most patients (72%) given thromboprophylaxis. (Low Molecular Weight Heparin)	-	-
14	Haksteen, Hilderink [20]	All patients received standard dose thromboprophylaxis. (Nadroparin)	-	-
15	Arribalzaga, Martinez-Alfonzo [21]	Most patients (68.3%) received standard prophylactic dose. (Low Molecular Weight Heparin)	Intermediate and therapeutic doses of LMWH were used more in ICU patients (18%) than in ward patients (12.6%).	-
16	Valle, Bonaffini [22]	91 patients received standard prophylactic dose.	-	-
17	Silva, Jorge [27]	29 patients received standard prophylactic dose.	-	-
18	Whyte, Kelly [42]	All patients given thromboprophylaxis in the absence of contraindication	-	-
19	Bruggemann, Spaetgens [23]	Most patients (55%) received standard prophylactic dose.	-	-
20	Chang, Rockman [34]	-	Most patients (62.2%) received therapeutic dose.	-
21	Chaudhary, Padrnos [35]	Most patients (80.4%) received standard prophylactic dose.	-	-
22	Fujiwara, Nakajima [38]	Only 10% received standard prophylactic dose. Mostly in ICU (*n* = 20/35 patients)	-	-
23	Lodigiani, Iapichino [25]	Thromboprophylaxis was used in 100% of ICU patients and 75% of those on the general ward.	-	-
24	Vivan, Rigatti [38]	68% of patients were receiving prophylactic or therapeutic doses. (Heparin)		-
25	Fraissé, Logre [26]	All patients received usual (prophylactic) or full-dose (therapeutic) anticoagulation according to their risk factors for thrombosis		-

The incidence of every reported outcome suggests that the management of TE complications used in all studies may be the cause of potential discrepancies. The management of thromboprophylaxis and therapeutic strategies involving antiplatelet or anticoagulant differed according to the study protocol and local guidelines. A post-mortem examination done in seven COVID-19 patients found platelet-rich thrombi in several organs, such as the pulmonary, hepatic, renal, and cardiac microvasculature [60]. From this finding, we can postulate that the beneficial effect of antiplatelets such as acetylsalicylic acid (ASA) had the advantage of preventing microthrombi in COVID-19 patients [61]. Furthermore, several studies found ASA has a pleiotropic effect of disturbing virus replication on the endothelium cell, which may target the development of endotheliitis in COVID-19 patients [62, 63]. In situations involving endothelial damage whereby the platelets stick to the injured site, causing thrombosis, antiplatelets such as ASA will be relevant in prophylactic treatment in preventing platelets from clumping together, hence causing TE complications [64]. However, ASA is also known for its bleeding complication, hence making it a contraindication for patients with an existing risk of bleeding.

On the other hand, anticoagulants such as heparin, low molecular-weight heparin (LMWH), or unfractionated heparin (UFH) have anti-inflammatory properties due to their ability to inhibit the formation of thrombin and reduce inflammatory responses [65]. Moreover, its antiviral potency explains the prevention of COVID-19 viral entry by acting on the angiotensin-converting enzyme 2 receptor and interacting with COVID-19 spike glycoprotein [61]. Despite its advantages of being pluripotent in nature, patients may develop heparin resistance and may need close monitoring of some parameters such as antithrombin activity, platelet count, factor VII, and fibrinogen level [63].

Several studies compared the outcomes of COVID-19 infection severity or mortality in patients receiving anticoagulant prophylactic dose versus anticoagulant therapeutic dose in hospitalized COVID-19 patients [66–71]. Most of the intervention studies found no significant outcomes in both prophylactic and therapeutic groups. An intervention study performed in Brazil compared the prophylactic regime (enoxaparin or UFH) and therapeutic dose (rivaroxaban: stable patients and enoxaparin or UFH: unstable patients). The result of this study found no significant beneficial effect of prophylactic over therapeutic regimes in terms of mortality or length of hospital stay. Instead, there was a significant increase in bleeding events in the therapeutic cohort (8% vs. 2%, *p* = 0.0010) [69]. The result was further supported by another intervention study conducted in critically ill hospitalized COVID-19 patients, which found the therapeutic dose of heparin showed no significant superiority in reducing mortality compared to the prophylactic group (OR 0.84; 95 CI: 0.64–1.11) [70]. Another multicentre randomized trial involving 28 hospitals in 6 countries among moderately ill COVID-19 patients with elevated d-dimer compared the standard prophylactic heparin dose with the standard therapeutic dose [66]. This study found that the therapeutic group did not show any significant association with a reduction of the primary composite of death, mechanical ventilation, or ICU admission compared with prophylactic heparin (OR: 0.69, 95% CI: 0.43–1.10, *P* = 0.12).

In contrast, a multicenter randomized clinical trial done by Spyropoulos, Goldin [67] found that within non-critically ill hospitalized COVID-19 patients, the therapeutic dose (enoxaparin) was associated with a reduction in TE complications (RR, 0.37; 95% CI, 0.21–0.66; *P* < 0.001) and a reduction in mortality at 28 days of hospitalization (relative risk (RR), 0.68; 95% CI, 0.49–0.96; *p* = 0.03). However, the result was different in critically ill COVID-19 patients in the ICU as the primary outcome, which showed no significant difference in TE complications in both groups (RR 0.92; 95% CI, 0.62–1.39; *p* = 0.71). Hence, we can presume that the condition of the patient played an important factor in determining which group had a superior beneficial effect.

In addition, the wide range of mortality (12.5-63.1%) [14, 28] in critically ill COVID-19 patients may be due to variations in heparin administration and thromboprophylaxis management. According to a study [72], the incidence of mortality was high in COVID-19 patients with elevated D-dimer levels who did not receive any thromboprophylaxis treatment. Researchers further supported this results by finding that both therapeutic and prophylactic anticoagulant regimens were associated with a reduction in in-hospital mortality compared to patients without anticoagulants [51].

In addition to the benefit of prophylaxis management in hospitalized COVID-19 patients, researchers in every study need to consider the risk of bleeding in their study populations. This is crucial, as every patient started on an anticoagulant may encounter the risk of hemorrhage. A study conducted by [51] found that some patients experienced bleeding events after the initiation of anticoagulant treatment. Patients who started on therapeutic doses experienced a higher rate of bleeding compared to those who did not receive any anticoagulants.

Although the episodes of bleeding complications were comparable in both the prophylaxis and therapeutic-dose groups [71], there was a difference in intensity depending on the type of anticoagulant used. For example, patients who were given a single preventative agent had higher bleeding rates when taking unfractionated heparin (UFH) than when taking low molecular weight heparin (LMWH). On the other hand, patients who were given therapeutic agents had higher bleeding rates when taking LMWH than when taking direct oral anticoagulants (DOACs) [51].

Finally, the limitations of this study should be considered. Although there was no duplication in the selected articles, there may be unintended bias due to the absence of registration in the PROPERO system.

## Conclusions

Overall, there was a wide range of incidences of both VTE complications and ATE complications among hospitalized COVID-19 patients (VTE: 0.4-84% and ATE: 0.5-15.2%). Similarly, a wide variation in the incidence between all-cause mortality in COVID-19 and the incidence of mortality associated with TE complications was seen in hospitalized COVID-19 patients (all-cause mortality:4.8-63.1% and mortality associated with TE complications:5.3-48.6%). These discrepancies may be the result of different definitions, diagnostic methods, and prophylaxis management across all the included studies. Multinational, multicenter data included in this review summarized the common occurrence of TE complications and associated mortality in COVID-19 patients. Therefore, every patient should undergo a thorough risk factor assessment for TE complications and allow individualized optimal thromboprophylaxis management to improve the patient’s outcome.

## Data Availability

All data have been included in the manuscript.

## References

[1] Li Q, Guan X, Wu P, Wang X, Zhou L, Tong Y et al. Early transmission dynamics in Wuhan, China, of novel coronavirus–infected pneumonia. N Engl J Med. 2020. 10.1056/NEJMoa2001316 PMC7121484 31995857

[2] Ferrario CM, Jessup J, Chappell MC, Averill DB, Brosnihan KB, Tallant EA (2005). Effect of angiotensin-converting enzyme inhibition and angiotensin II receptor blockers on cardiac angiotensin-converting enzyme 2. Circulation.

[3] Liu Y, Zhang H-G (2021). Vigilance on new-onset atherosclerosis following SARS-CoV-2 infection. Front Med.

[4] Adhikari SP, Meng S, Wu Y-J, Mao Y-P, Ye R-X, Wang Q-Z (2020). Epidemiology, causes, clinical manifestation and diagnosis, prevention and control of coronavirus disease (COVID-19) during the early outbreak period: a scoping review. Infect Dis Poverty.

[5] Nägele MP, Haubner B, Tanner FC, Ruschitzka F, Flammer AJ (2020). Endothelial dysfunction in COVID-19: current findings and therapeutic implications. Atherosclerosis.

[6] Hattori Y, Hattori K, Machida T, Matsuda N (2022). Vascular endotheliitis associated with infections: its pathogenetic role and therapeutic implication. Biochem Pharmacol.

[7] Ribes A, Vardon-Bounes F, Mémier V, Poette M, Au-Duong J, Garcia C (2020). Thromboembolic events and Covid-19. Adv Biol Regul.

[8] Avila J, Long B, Holladay D, Gottlieb M (2021). Thrombotic complications of COVID-19. Am J Emerg Med.

[9] Thomas W, Varley J, Johnston A, Symington E, Robinson M, Sheares K (2020). Thrombotic complications of patients admitted to intensive care with COVID-19 at a teaching hospital in the United Kingdom. Thromb Res.

[10] Zhang L, Feng X, Zhang D, Jiang C, Mei H, Wang J (2020). Deep vein thrombosis in hospitalized patients with COVID-19 in Wuhan, China: prevalence, risk factors, and outcome. Circulation.

[11] Gue YX, Tennyson M, Gao J, Ren S, Kanji R, Gorog DA (2020). Development of a novel risk score to predict mortality in patients admitted to hospital with COVID-19. Sci Rep.

[12] Wang Q, Zennadi R (2020). Oxidative stress and thrombosis during aging: the roles of oxidative stress in RBCs in venous thrombosis. Int J Mol Sci.

[13] Zhou F, Yu T, Du R, Fan G, Liu Y, Liu Z (2020). Clinical course and risk factors for mortality of adult inpatients with COVID-19 in Wuhan, China: a retrospective cohort study. Lancet.

[14] Klok F, Kruip M, Van der Meer N, Arbous M, Gommers D, Kant K (2020). Incidence of thrombotic complications in critically ill ICU patients with COVID-19. Thromb Res.

[15] Al-Samkari H, Karp Leaf RS, Dzik WH, Carlson JCT, Fogerty AE, Waheed A (2020). COVID-19 and coagulation: bleeding and thrombotic manifestations of SARS-CoV-2 infection. Blood.

[16] Mohamud MFY, Mukhtar MS (2022). Epidemiological characteristics, clinical relevance, and risk factors of thromboembolic complications among patients with COVID-19 pneumonia at a teaching hospital: retrospective observational study. Annals Med Surg.

[17] Kaptein FHJ, Stals MAM, Grootenboers M, Braken SJE, Burggraaf JLI, van Bussel BCT (2021). Incidence of thrombotic complications and overall survival in hospitalized patients with COVID-19 in the second and first wave. Thromb Res.

[18] Gonzalez-Fajardo JA, Ansuategui M, Romero C, Comanges A, Gomez-Arbelaez D, Ibarra G (2021). Mortality of COVID-19 patients with vascular thrombotic complications. Med Clin (Engl Ed).

[19] Jimenez-Guiu X, Huici-Sanchez M, Rmera-Villegas A, Izquierdo-Miranda A, Sancho-Cerro A, Vila-Coll R (2021). Deep vein thrombosis in noncritically ill patients with coronavirus disease 2019 pneumonia: deep vein thrombosis in nonintensive care unit patients. J Vasc Surg Venous Lymphat Disord.

[20] García-Ortega A, Oscullo G, Calvillo P, López-Reyes R, Méndez R, Gómez-Olivas JD (2021). Incidence, risk factors, and thrombotic load of pulmonary embolism in patients hospitalized for COVID-19 infection. J Infect.

[21] Chen B, Jiang C, Han B, Guan C, Fang G, Yan S (2021). High prevalence of occult thrombosis in cases of mild/moderate COVID-19. Int J Infect Dis.

[22] Martinot M, Eyriey M, Gravier S, Bonijoly T, Kayser D, Ion C (2021). Predictors of mortality, ICU hospitalization, and extrapulmonary complications in COVID-19 patients. Infect Dis Now.

[23] Munoz-Rivas N, Abad-Motos A, Mestre-Gomez B, Sierra-Hidalgo F, Cortina-Camarero C, Lorente-Ramos RM (2021). Systemic thrombosis in a large cohort of COVID-19 patients despite thromboprophylaxis: a retrospective study. Thromb Res.

[24] Kajoak S, Osman H, Elnour H, Elzaki A, Alghamdi AJ, Elhaj M (2022). The prevalence of pulmonary embolism among COVID-19 patients underwent CT pulmonary angiography. J Radiation Res Appl Sci.

[25] Tholin B, Fiskvik H, Tveita A, Tsykonova G, Opperud H, Busterud K et al. Thromboembolic complications during and after hospitalization for COVID-19: incidence, risk factors and thromboprophylaxis. Thromb Update. 2022:100096. 10.1016/j.tru.2021.100096 PMC8720677 38620916

[26] Martínez Chamorro E, Revilla Ostolaza TY, Pérez Núñez M, Borruel Nacenta S, Cruz-Conde Rodríguez-Guerra C, Ibáñez Sanz L (2021). Pulmonary embolisms in patients with COVID-19: a prevalence study in a tertiary hospital. Radiología (English Edition).

[27] Bozzani A, Arici V, Tavazzi G, Franciscone MM, Danesino V, Rota M (2020). Acute arterial and deep venous thromboembolism in COVID-19 patients: risk factors and personalized therapy. Surgery.

[28] Elboushi A, Syed A, Pasenidou K, Elmi L, Keen I, Heining C et al. Arterial and venous thromboembolism in critically ill, COVID 19 positive patients admitted to Intensive Care Unit. Ann Vasc Surg. 2022. 10.1016/j.avsg.2022.02.005 PMC8894740 35257911

[29] Rali P, O’Corragain O, Oresanya L, Yu D, Sheriff O, Weiss R (2021). Incidence of venous thromboembolism in coronavirus disease 2019: an experience from a single large academic center. J Vasc Surg Venous Lymphat Disord.

[30] Erben Y, Franco-Mesa C, Gloviczki P, Stone W, Quinones-Hinojoas A, Meltzer AJ (2021). Deep vein thrombosis and pulmonary embolism among hospitalized coronavirus disease 2019-positive patients predicted for higher mortality and prolonged intensive care unit and hospital stays in a multisite healthcare system. J Vasc Surg Venous Lymphat Disord.

[31] Filippi L, Sartori M, Facci M, Trentin M, Armani A, Guadagnin ML (2021). Pulmonary embolism in patients with COVID-19 pneumonia: when we have to search for it?. Thromb Res.

[32] Brandao A, de Oliveira CZ, Rojas SO, Ordinola AAM, Queiroz VM, de Farias DLC (2021). Thromboembolic and bleeding events in intensive care unit patients with COVID-19: results from a Brazilian tertiary hospital. Int J Infect Dis.

[33] Haksteen WE, Hilderink BN, Dujardin RWG, Jansen RR, Hodiamont CJ, Tuinman PR (2021). Venous thromboembolism is not a risk factor for the development of bloodstream infections in critically ill COVID-19 patients. Thromb Res.

[34] Arribalzaga K, Martinez-Alfonzo I, Diaz-Aizpun C, Gutierrez-Jomarron I, Rodriguez M, Castro Quismondo N (2021). Incidence and clinical profile of venous thromboembolism in hospitalized COVID-19 patients from Madrid region. Thromb Res.

[35] Valle C, Bonaffini PA, Dal Corso M, Mercanzin E, Franco PN, Sonzogni A (2021). Association between pulmonary embolism and COVID-19 severe pneumonia: experience from two centers in the core of the infection Italian peak. Eur J Radiol.

[36] Silva BV, Jorge C, Placido R, Mendonca C, Urbano ML, Rodrigues T (2021). Pulmonary embolism and COVID-19: a comparative analysis of different diagnostic models performance. Am J Emerg Med.

[37] Whyte MB, Kelly PA, Gonzalez E, Arya R, Roberts LN (2020). Pulmonary embolism in hospitalised patients with COVID-19. Thromb Res.

[38] Vivan MA, Rigatti B, da Cunha SV, Frison GC, Antoniazzi LQ, de Oliveira PHK (2022). Pulmonary embolism in patients with COVID-19 and D-dimer diagnostic value: a retrospective study. Braz J Infect Dis.

[39] Bruggemann RAG, Spaetgens B, Gietema HA, Brouns SHA, Stassen PM, Magdelijns FJ (2020). The prevalence of pulmonary embolism in patients with COVID-19 and respiratory decline: a three-setting comparison. Thromb Res.

[40] Chang H, Rockman CB, Jacobowitz GR, Speranza G, Johnson WS, Horowitz JM (2021). Deep vein thrombosis in hospitalized patients with coronavirus disease 2019. J Vasc Surg Venous Lymphat Disord.

[41] Chaudhary R, Padrnos L, Wysokinska E, Pruthi R, Misra S, Sridharan M (2021). Macrovascular thrombotic events in a Mayo Clinic enterprise-wide sample of hospitalized COVID-19-Positive compared with COVID-19-Negative patients. Mayo Clin Proc.

[42] Fujiwara S, Nakajima M, Kaszynski RH, Fukushima K, Tanaka M, Yajima K (2021). Prevalence of thromboembolic events and status of prophylactic anticoagulant therapy in hospitalized patients with COVID-19 in Japan. J Infect Chemother.

[43] Helms J, Tacquard C, Severac F, Leonard-Lorant I, Ohana M, Delabranche X (2020). High risk of thrombosis in patients with severe SARS-CoV-2 infection: a multicenter prospective cohort study. Intensive Care Med.

[44] Lodigiani C, Iapichino G, Carenzo L, Cecconi M, Ferrazzi P, Sebastian T (2020). Venous and arterial thromboembolic complications in COVID-19 patients admitted to an academic hospital in Milan, Italy. Thromb Res.

[45] Cueto-Robledo G, Navarro-Vergara D-I, Roldan-Valadez E, Garcia-Cesar M, Graniel-Palafox L-E, Cueto-Romero H-D et al. Pulmonary embolism (PE) prevalence in mexican-mestizo patients with severe SARS-COV-2 (COVID-19) pneumonia at a tertiary-level hospital: a review. Curr Probl Cardiol. 2022:101208. 10.1016/j.cpcardiol.2022.101208 PMC9020648 35460689

[46] Fraissé M, Logre E, Pajot O, Mentec H, Plantefève G, Contou D (2020). Thrombotic and hemorrhagic events in critically ill COVID-19 patients: a French monocenter retrospective study. Crit Care.

[47] Choudhary S, Sharma K, Singh PK (2021). Von Willebrand factor: a key glycoprotein involved in thrombo-inflammatory complications of COVID-19. Chemico-Biol Interact.

[48] Tan Y-K, Goh C, Leow AS, Tambyah PA, Ang A, Yap E-S (2020). COVID-19 and ischemic stroke: a systematic review and meta-summary of the literature. J Thromb Thrombolysis.

[49] Roberts LN, Whyte MB, Georgiou L, Giron G, Czuprynska J, Rea C (2020). Postdischarge venous thromboembolism following hospital admission with COVID-19. Blood.

[50] Engelen MM, Vandenbriele C, Balthazar T, Claeys E, Gunst J, Guler I, et al. editors. Venous thromboembolism in patients discharged after COVID-19 hospitalization. Seminars in thrombosis and hemostasis. Thieme Medical Publishers, Inc.; 2021. 10.1055/s-0041-1727284 33893631

[51] Nadkarni GN, Lala A, Bagiella E, Chang HL, Moreno PR, Pujadas E (2020). Anticoagulation, bleeding, mortality, and pathology in hospitalized patients with COVID-19. J Am Coll Cardiol.

[52] Garcia-Ortega A, Oscullo G, Calvillo P, Lopez-Reyes R, Mendez R, Gomez-Olivas JD (2021). Incidence, risk factors, and thrombotic load of pulmonary embolism in patients hospitalized for COVID-19 infection. J Infect.

[53] Chaudhry R, Miao JH, Rehman A, Physiology. cardiovascular. StatPearls [Internet]: StatPearls Publishing; 2022. 29630249

[54] Schofield Z, Baksamawi HA, Campos J, Alexiadis A, Nash GB, Brill A (2020). The role of valve stiffness in the insurgence of deep vein thrombosis. Commun Mater.

[55] López JA, Kearon C, Lee AY (2004). Deep venous thrombosis. ASH Educ Program Book.

[56] Abou-Ismail MY, Diamond A, Kapoor S, Arafah Y, Nayak L (2020). The hypercoagulable state in COVID-19: incidence, pathophysiology, and management. Thromb Res.

[57] Tholin B, Ghanima W, Einvik G, Aarli B, Brønstad E, Skjønsberg OH (2021). Incidence of thrombotic complications in hospitalised and non-hospitalised patients after COVID-19 diagnosis. Br J Haematol.

[58] Alizadehsani R, Alizadeh Sani Z, Behjati M, Roshanzamir Z, Hussain S, Abedini N (2021). Risk factors prediction, clinical outcomes, and mortality in COVID-19 patients. J Med Virol.

[59] Kaptein F, Stals M, Huisman M, Klok F (2021). Prophylaxis and treatment of COVID-19 related venous thromboembolism. Postgrad Med.

[60] Rapkiewicz AV, Mai X, Carsons SE, Pittaluga S, Kleiner DE, Berger JS et al. Megakaryocytes and platelet-fibrin thrombi characterize multi-organ thrombosis at autopsy in COVID-19: a case series. EClinicalMedicine. 2020;24. 10.1016/j.eclinm.2020.100434 PMC7316051 32766543

[61] Santoro F, Núñez-Gil IJ, Vitale E, Viana‐Llamas MC, Romero R, Maroun Eid C (2022). Aspirin therapy on prophylactic anticoagulation for patients hospitalized with COVID‐19: a propensity score‐matched cohort analysis of the HOPE‐COVID‐19 Registry. J Am Heart Association.

[62] Florêncio FKZ, Tenório MO, Macedo Júnior ARA, Lima SG (2020). Aspirin with or without statin in the treatment of endotheliitis, thrombosis, and ischemia in coronavirus disease. Rev Soc Bras Med Trop.

[63] Khandelwal G, Ray A, Sethi S, Harikrishnan H, Khandelwal C, Sadasivam B (2021). COVID-19 and thrombotic complications—the role of anticoagulants, antiplatelets and thrombolytics. J Family Med Prim Care.

[64] Krötz F, Sohn H-Y, Klauss V (2008). Antiplatelet drugs in cardiological practice: established strategies and new developments. Vasc Health Risk Manag.

[65] Hippensteel JA, LaRiviere WB, Colbert JF, Langouët-Astrié CJ, Schmidt EP (2020). Heparin as a therapy for COVID-19: current evidence and future possibilities. Am J Physiology-Lung Cell Mol Physiol.

[66] Sholzberg M, Tang GH, Rahhal H, AlHamzah M, Kreuziger LB, Áinle FN et al. Effectiveness of therapeutic heparin versus prophylactic heparin on death, mechanical ventilation, or intensive care unit admission in moderately ill patients with covid-19 admitted to hospital: RAPID randomised clinical trial. BMJ. 2021;375. 10.1136/bmj.n2400 PMC8515466 34649864

[67] Spyropoulos AC, Goldin M, Giannis D, Diab W, Wang J, Khanijo S (2021). Efficacy and safety of therapeutic-dose heparin vs standard prophylactic or intermediate-dose heparins for thromboprophylaxis in high-risk hospitalized patients with COVID-19: the HEP-COVID randomized clinical trial. JAMA Intern Med.

[68] Marcos-Jubilar M, Carmona-Torre F, Vidal R, Ruiz-Artacho P, Filella D, Carbonell C (2022). Therapeutic versus prophylactic bemiparin in hospitalized patients with nonsevere COVID-19 pneumonia (BEMICOP study): an open-label, multicenter, randomized, controlled trial. Thromb Haemost.

[69] Lopes RD, Furtado RH, Macedo AVS, Bronhara B, Damiani LP, Barbosa LM (2021). Therapeutic versus prophylactic anticoagulation for patients admitted to hospital with COVID-19 and elevated D-dimer concentration (ACTION): an open-label, multicentre, randomised, controlled trial. Lancet.

[70] Godoy LC, Neal MD, Goligher EC, Cushman M, Houston BL, Bradbury CA (2024). Heparin dose intensity and organ support-free days in patients hospitalized for COVID-19. JACC: Adv.

[71] REMAP-CAP A-a, Investigators A (2021). Therapeutic anticoagulation with heparin in critically ill patients with Covid-19. N Engl J Med.

[72] Tang N, Bai H, Chen X, Gong J, Li D, Sun Z (2020). Anticoagulant treatment is associated with decreased mortality in severe coronavirus disease 2019 patients with coagulopathy. J Thromb Haemost.

